# Preliminary validity and reliability evidence of the Brief Antisocial Behavior Scale (B-ABS) in young adults from four countries

**DOI:** 10.1371/journal.pone.0247528

**Published:** 2021-02-22

**Authors:** Laura Mezquita, Adrian J. Bravo, Angelina Pilatti, Generós Ortet, Manuel I. Ibáñez, Cross-Cultural Addictions Study Team

**Affiliations:** 1 Department of Basic and Clinical Psychology and Psychobiology, Universitat Jaume I, Castellón de la Plana, Castellón, Spain; 2 Centre for Biomedical Research Network on Mental Health (CIBERSAM), Instituto de Salud Carlos III, Castellón de la Plana, Castellón, Spain; 3 Department of Psychological Sciences, William & Mary, Williamsburg, Virginia, United States of America; 4 Facultad de Psicología, Universidad Nacional de Córdoba, CIPSI Grupo Vinculado CIECS-UNC-CONICET, Córdoba, Córdoba, Argentina; University of La Rioja, SPAIN

## Abstract

The present research built on the Self-Reported Delinquency interview and the Antisocial Behavior Scale to develop an updated brief instrument to measure antisocial behavior. College students (*n* = 3188, 67.75% women) from the USA, Argentina, the Netherlands and Spain completed an online survey. Analyses that combined approaches from the Classical Test Theory and Item Response Theory were conducted to select the items for the brief version. Findings suggested that a 13-item Brief Antisocial Behavior Scale (B-ABS) fulfilled the high-quality criteria: salient factor loadings, adequate discrimination, variability in response endorsement, adequate fit based on infit/outfit values, nondifferent item functioning across the four participating countries, and Cronbach’s alpha and ordinal omega coefficients higher than .70. The B-ABS scores generally significantly correlated with personality scores, mental health and marijuana outcomes, showing criterion-related validity evidence. Our overall findings suggest that B-ABS adequately assesses antisocial behavior in young adults from different countries/cultures.

## Introduction

Antisocial behavior is broadly defined as actions that violate societal norms and others’ personal or property rights [1]. Antisocial behavior begins in early adolescence and is related to higher risks of both criminal justice involvement and premature death [2, 3]. Antisocial behavior usually peaks in late adolescence or the beginning of adulthood showing a declining pattern after this life period [4]. In addition, some youths will show a persistent pattern of antisocial behavior throughout their lifetime [5, 6]. In fact in the USA, recent prevalence rates suggest that one in 4 US adults exhibit syndromal antisocial behavior, and antisocial syndromes are associated with other psychiatric disorders [e.g., bipolar I, posttraumatic stress disorder, borderline personality disorder, and schizotypal personality disorders; 7]. In young adults, antisocial behavior has been related to psychological distress (including symptoms of somatization, depression, anxiety, phobic anxiety, interpersonal sensitivity, obsessive-compulsivity, hostility, paranoid ideation, and psychoticism) [8], depression [9], alcohol use [10, 11], and illicit drugs use [12], among others. A recent meta-analysis has also showed that antisocial personality disorder has a prevalence of 3.05%. However, the majority of the studies included preceded from USA [13], suggesting the need of performing similar epidemiologic studies in other countries around the world.

To know whether a psychological phenomenon occurs across cultures is important for understanding the interplay between individuals and their cultural context [14, 15]. In this line of research, previous studies have suggested that rates of antisocial behavior and related symptoms appear to be more common in individualistic and industrialized cultures than in collectivistic cultures, that is in cultures that prioritize the needs of an individual over the needs of a group as a whole [15, 16]. However, other studies did not identify this pattern clearly, and suggest that income inequality could be responsible of higher antisocial behavior and violence by country [17, 18]. In any case, due to the lack of cross-national studies, further research is needed to better understand the role of the cultural context in the antisocial behavior. The scare number of these types of studies is in part due to the lack of equivalent measures to assess such behavior across different languages and nations.

### History of the antisocial behavior scale

The Self-Reported Delinquency Interview [SRD; 19] was developed to assess antisocial behavior, understood as the transgression of laws against people and property, and as the violation of norms that imply common administrative sanctions in western societies. The original SRD contained 48 questions, administrated to boys (aged between 11–12 years) who were followed up 2 years later. The principal component analysis of the interview suggested the existence of a general factor of delinquency that underlay the structure of the questionnaire.

Based on Shapland’s [19] interview to assess antisocial behavior in boys aged 11–14 years, Pérez-Sánchez and Torrubia [20] translated and adapted items into Spanish, and also adapted the interview to be administrated as a self-report measure. First, they omitted 13 non-delinquent (e.g., “I have been fishing” or “I have been skating”) and added three mischievous (e.g., “I have dialed 999 just for joke) items from Shapland’s original version of 48 items. Second, they reworded three items that could reflect antisocial behavior in youngsters, but not in older people (e.g., “I have smoked cigarettes” to “I smoked cigarettes before the age of 16”). Finally, they added five items: “I have sprayed a message on a wall”, “I have used illegal gadgets to make a telephone call”, “I have broken the highway code (by failing to stop when lights are red or failing to respect other drivers’ right of way, etc.), “I have had a firearm in my possession for reasons other than for sport or for strict military use” and “I have obtained money through drug-trafficking”. The resulting Spanish SRD [S-SRD; 20] consists of a 37-item scale in which participants report whether they endorse (or not) each item (i.e., a yes/no scale) and a frequency score (i.e., with two response options: from 1 to 6 times, or 6 times or more). The authors showed that the sum of the total number of endorsed items correlated with sensation seeking in their sample of male and female students [20].

In a more recent study examining different etiological pathways to alcohol use and misuse, the Spanish SRD was revamped [21]. As the measure aimed to assess young adults in addition to adolescents, the name of the scale was modified to the Antisocial Behavior Scale (ABS), rather than using the term delinquency. In addition, when considering that the original scale was developed to assess adolescents aged from 11 to 14 years in order to assess more antinormative behaviors, the temporal specifications of some items changed from 16 or 18 years old to 14 years old (e.g., “I smoked cigarettes before the age of 14”). Furthermore, six items that were not considered antinormative in the Spanish socio-cultural context (e.g., “I have let off fireworks in the street”), or were old-fashioned (e.g., I have stolen school property worth more than about 100 pesetas) were deleted. Finally, four other items, including some related to the DSM and ICD criteria to diagnose Conduct Disorder, were included (e.g., I have mistreated animals). The response scale was also modified, and a 4-point scale (from *never or almost never* to *very frequently*) was employed rather than dichotomous items. The internal consistency of the scale was adequate (α = .88) and criterion-related validity evidence was shown. Specifically, the personality traits of (low) agreeableness and (low) conscientiousness were related to the antisocial behavior scores five years later, even when gender, age and previous alcohol use were controlled for, and antisocial behavior cross-sectionally predicted alcohol-related variables [21].

Although the scale has been shown sources of validity and reliability evidence, a 35-item scale may be too long for research endeavors in which assessment is time-limited or when the evaluation requires administering several tests/measures. The development of very brief measures is especially useful in cross-cultural research, in which the cost in terms of organization, coordination, and monetary price is very high. Therefore, there is a need, to obtain efficient instruments insofar as how much information they provide and the amount of time is needed to answer them; that is, one that is balanced in validity and utility terms [22].

### Present study

A variety of measures assess antisocial behavior [e.g., the Antisocial Processes Screening Device; 23, Psychopathy Checklist-Revised; 24, A-D. Cuestionario de Conductas Antisociales-Delictivas; 25], but very few have been psychometrically tested to account for cultural differences. What is “normative behavior” varies across distinct cultures, nationalities and regions [see 26], and most psychological constructs are highly dependent on the cultural context in which tests are used. Therefore, a central aspect to test development is to determine whether measures operate in the same way across languages or groups of administration. Furthermore, comparisons are not valid until multicultural and multilanguage research confirms measurement equivalence [27]. The present study aimed to develop a brief version of the ABS (B-ABS) to assess antisocial behavior in two individualistic (USA and the Netherlands) and two collectivistic countries (Argentina and Spain). To accomplish this goal, a set of analyses based on Classical Test Theory (discrimination index and difficulty indices) and Item Response Theory (i.e., Rasch analysis, including differential item functioning analysis) were conducted. We expected that the resulting B-ABS shows evidence of reliability of its scores across countries (i.e., Cronbach’s alphas and Ordinal omega of .70 or higher). Additionally, associations between the B-ABS and different criterion-related variables (e.g., personality traits, mental health symptoms, and marijuana-related variables) were estimated to provide sources of criterion-related validity evidence of the scale. Specifically, we hypothesized that low agreeableness, low conscientiousness [28–30], impulsivity traits [31] and psychopathological symptoms would be related to the antisocial behavior [7–12]. We also expect higher correlations between the antisocial behavior and the drug use variables than with internalizing-related symptoms, as they are considered part of the same spectrum [32, 33]. Due to the lack of similar cross-national research, the study of the differences in the magnitude of these associations across countries are exploratory.

## Method

### Participants and procedures

The original sample included 3349 college students from four universities of USA (*n* = 1928, 66.89% females, Mean age = 19.95, *SD =* 4.16), one from Argentina (*n* = 375, 66.67% females, Mean age = 24.16, *SD =* 5.38), one from the Netherlands (*n* = 302, 73.51% females, Mean age = 20.87, *SD =* 2.96) and one from Spain (*n* = 754, 66.45% females, Mean age = 21.44, *SD =* 3.99), who completed an online survey study about personal mental health, personality traits and marijuana use behaviors [for more information, see 34]. For the USA sites, students were recruited from Psychology Department research pools and received research credit for their participation. In Argentina students were recruited through online social networks and email listings and entered into a raffle of seven prizes [one stay in a cottage located in a popular destination and six cash prizes (each of ≈US 36 at the time)]. In Spain, an email was sent to all the students at the university inviting them to participate in the research. Participants who completed the survey received €5 for their participation. In the Netherlands, students were recruited from the School of Social and Behavioral Sciences and received research credit. The study was approved by the institutional review board (or their international equivalent) at each participating university (USA single site IRB for all U.S. sites from the University of New Mexico [Approval Code: 112900–2]; the Tilburg University approval reference: EC-2018.03; Argentina and Spain resolution data: 12/13/2017 and 3/29/2018, respectively). All the participants completed the informed consent in the first page of the online survey.

For the present study, only the data of the students that completed the ABS were included. After excluding the individuals with more than 5% of missing values in the ABS (*n* = 63), and who did not disclose their gender (*n* = 13), the total analytic sample was composed of 3188 participants (USA: *n* = 1873, 67.49% females, mean age = 19.87, *SD =* 4.20); Argentina: *n* = 346, 65.61% females, mean age = 24.31, *SD =* 5.49); the Netherlands: *n* = 294, 74.49% females, mean age = 20.79, *SD =* 2.76); Spain, *n* = 675, 66.67% females, mean age = 21.41, *SD =* 3.95).

### Measurement translation and adaptation

As the international guidelines for test adaptations suggested [35], an iterative procedure to develop the different versions of the questionnaires across languages was followed. In a previous project [see 36 for more information], two psychologists proficient in English and Spanish and with expertise in test adaptation and addictive behaviors translated the most updated version of the scale, the ABS [21], into English. Then a bilingual teacher unfamiliar with the inventory did a back translation. The analysis of the back translation indicated that the English version could be considered comparable to the original scale. Some minor changes were made in the Castilian Spanish version of the ABS to ensure that all the items were adequate for the Argentinian participants. The same procedure was followed in the project of which this study forms part to adapt the marijuana outcomes from English to Spanish [see 34 for more details]. To adapt the measure to Dutch, research team members, who are bicultural and proficient in English, Spanish and Dutch, with expertise in test adaptations, translated the original English/Spanish versions of the ABS, the personality questionnaires, the mental health measure and the marijuana outcomes questionnaires into Dutch (see below for a detailed description of the scales). Then the research team members compared the versions and, after a thorough discussion, composed a preliminary version of the instruments. Items from the English, Dutch and Spanish ABS versions are presented in S1 Table.

### Measures

#### Antisocial behavior

The ABS [21] includes 35 items that describe different antisocial behaviors (e.g. “I have used knives or sticks in fights” or “I have broken, ripped or damaged public properties”) on a 4-point response scale (1 = *never or almost never*, 2 = *sometimes*, 3 = *frequently*, 4 = *very frequently or very often*). The total score is obtained by summing the responses to all the items. A previous preliminary study showed that the scores for the English and Spanish ABS versions had good internal consistency. The different item functioning analyses showed that the items generally operate similarly across the USA, Argentina and Spain [36].

#### Personality traits

Personality traits were assessed by the 50-item *Big Five Personality Trait Short Questionnaire* [BFPTSQ; 37] at the US sites, the Spanish version [38] at the sites in Spain and Argentina, and our own adaptation to Dutch in the Netherlands. The measure assessed the Five-Factor Model (FFM) broad personality domains on a 5-point Likert response scale (0 = *strongly disagree*, 4 = *strongly agree*): *openness*, *extraversion*, *agreeableness*, *conscientiousness*, and *emotional stability*. Items were averaged, thus scores ranged from 0 to 4 in the present study. Recent research has tested the measurement invariance across languages and cultures of the scale, and found that the BFPTSQ showed configural, metric and scalar invariance among Argentinian and Spanish youths and between the English and the Spanish versions of the questionnaire [39].

#### Short UPPS-P

A 20-item English version of the UPPS-P Impulsive Behavior Scale [S UPPS-P; 40] was used to measure impulsivity facets of negative urgency, lack of perseverance, lack of premeditation, sensation seeking and positive urgency. The same 20 items were selected from the Spanish [41] and the Dutch UPPS-P [items were adapted in the current project; see 42] questionnaire to assess impulsivity among Argentinean, Spanish and Dutch youths. The scale response scale went from 1 to 4.

#### Mental health symptoms

Past 2-week psychopathology was assessed by the 23-item DSM-5 Self-Rated Level 1 Cross-Cutting Symptoms Measure-Adult [43]. The Spanish version was administered to the Spanish-speaking students [44]. The measure was translated into Dutch for the Dutch-speaking students [see 45 for more datails]. Participants were asked, “In the past 2 weeks, how much (or how often) have you been bothered by the following problems?” and responded on a 5-point response scale (0 = *none*, *not at all*, 1 = *slight or rare*, *less than a day or two*; 2 = *mild*, *several days*; 3 = *moderate*, *more than half the days*, 4 = s*evere*, *nearly every day*). A score of 2 or more in most domains, except substance use (score of 1 or more), suggests clinically-relevant mental health problems [46]. The measure has been validated with clinical [46] and college student [47] samples.

The prevalence rates of potential symptom presentation for the 13 domains (percentages were averaged for the domains with multiple items) of the DSM-5 Cross-Cutting Symptoms Measure in our analytic sample were as follows: depression (33.12%), anger (38.34%), mania (24.25%), anxiety (24.44%), somatic distress (18.31%), suicidal ideation (10.45%), psychosis (4.56%), sleep disturbance (29.75%), memory problems (16.58%), repetitive thoughts and behaviors (12.05%), dissociation (16.01%), and personality functioning (22.68%). For substance use, we present the rates for each specific substance: alcohol use (31.19%), tobacco (21.93%), and illicit drug use (18.35%).

#### Marijuana use

The participants who reported using marijuana at least once in the previous month (i.e., last-month marijuana users) completed a visual guide showing different amounts of marijuana in grams. The same guide was used across countries. The Marijuana Use Grid [MUG; 48] was used to assess typical marijuana use. Participants reported the amount of grams they used in each 4-hour period on each typical week day (12p-4p on Monday, 4p-8p on Monday, etc.). All non-zero values, which reflect the number of time periods used in a typical week (possible range: 0–42), were added up to obtain an estimate of typical marijuana use frequency. By summing all the values, we obtained an estimate of the typical marijuana use quantity by reflecting the total number of grams used in a typical week. To address outliers, we winsorized quantity estimates > 3SDs above the mean.

#### Negative marijuana-related consequences

Negative marijuana-related consequences were assessed using the B-MACQ [49]. Each item was scored dichotomously to reflect the presence/absence of the marijuana-related problem in the past month (0 = *no*, 1 = *yes*). The total score reflects the total number of consequences that an individual has experienced in the last 30 days. Recent research has tested the measurement invariance across languages and cultures of the scale, and found that a 20-item version showed configural and scalar invariance among the USA, Argentina, Uruguay and Spain. However, the questionnaire gave a poor fit in a sample of young adults from the Netherlands [34]. After considering these results, the 20-item version was herein used.

### Statistical analysis

#### Missing data imputation

From the 3188 participants, the 125 participants who present some missing data in the ABS came from USA (115 missing values from 65.555 possible values [35 items x 1873 participants], 0.18%) and from the Netherlands (10 missing values from 10.290 possible values [35 items x 294 participants], 0.10%). In Argentina and Spain, electronic prompts for each missing response were given and, therefore, there were no missing values. After checking that these missing values were at random (the maximum percentage of missing values in one item was 0.6%, which is lower than the recommended cut-off of 2%), we estimated the EFA using the full information maximum likelihood (FIML), while pairwise deletion was used in the classical test theory and item response theory analysis. Considering the low number of missing values, the mean score of the B-ABS and the rest of the scales included in the study were computed to provide evidences of criterion validity.

#### Exploratory factor analysis

An exploratory factor analysis (EFA) was performed to: 1) explore the structure underlying the set of items; 2) select those items with the highest factor loadings for the brief version. By considering the response scale of the questionnaire, the items were analyzed as categorical variables and the WLSMV estimator was used. To evaluate the overall model fit, the model fit criteria suggested by Marsh, Hau and Wen [50] were used, including the Comparative Fit Index (CFI) >.90 (acceptable) > .95 (optimal), Tucker-Lewis Index (TLI) >.90 (acceptable) > .95 (optimal), Root Mean Square Error of Approximation (RMSEA) < .06, and Standardized Root Mean Square Residual (SRMR) < .08. These analyses were conducted with Mplus 7.4. [51].

#### Classical Test Theory and Item Response Theory analysis

Following Meyer’s recommendations [52], we combined analyses and procedures based on the Classical Test Theory (discrimination index and difficulty indices) and the Item Response Theory (Rasch analyses, including differential item functioning) to identify the best items for the brief scale. We specifically examined the indices of discrimination for each item, which indicates the correlation between each item and the total test score (i.e., correlation item-total). By considering the number of points on the Likert scales, the discrimination index should lie between .43 and .83. Secondly, to reflect the item endorsability, we performed the item difficulty index (i.e., mean of the whole sample on each item). Third, we applied the Rasch Model [53], which is one particular IRT model, to evaluate person-item outfit and infit, and to determine whether items function in similar ways across countries (i.e., differential item functioning [DIF]). For the infit/outfit analyses, we used the unweighted mean square (UMS) and the weighted mean square (WMS) fit statistics, respectively. In both cases, values between .80 and 1.20 are recommended, while values between .50 and 1.5 are considered still productive for measurements [52]. Then we conducted a DIF analysis across each pair of countries (e.g., Spain and USA to explore the possible differences in the antisocial behavior estimates independently obtained for the college students from each country. We calculated the magnitude of the differences for each item between groups using the standardized P-DIF (sP-DIF), which is the result of dividing the standardized mean difference (SMD) by the item score range. An sP-DIF value below .05 indicates that items function similarly across college students from each country pair [52]. In other words, the absence of DIF suggests that the probability of each person endorsing every item is not affected by the country/culture of origin. To choose the final pool of items, we also followed some theoretical considerations (i.e., avoid item content and other associated constructs overlapping, such as drug use, or qualitative students’ and evaluators’ comments about understanding of item contents) apart from statistical considerations. These analyses were performed with the jMetrik software [52].

#### Internal consistency and sources of criterion-related validity evidence

To examine the internal consistency of the brief version, we calculated Cronbach’s alpha (conducted with the SPSS 23 software) and the omega coefficient (conducted with Mplus 7.4). For both coefficients, values above .70 indicate adequate internal consistency. We conducted Pearson’s correlation analyses to evaluate the association between the total B-ABS score and personality traits, mental health outcomes and marijuana-related measures. These analyses were conducted in each country.

## Results

### Exploratory factor analysis

The EFA showed an optimal data fit for the one-factor model (χ^2^ (595) = 77574.50, CFI = .971, TLI = .969; RMSEA = .035, SRMR = .050). The inclusion of an additional factor did not significantly improve the model fit (ΔCFI = .007, ΔTLI = .006, ΔRMSEA = -.003), which supports the single factor structure. The factor loadings of the final factor solution were all salient (>.30) and significant at *p* < .05. These results are presented in Table 1.

**Table 1:** Results of the Classical Test Theory and Item Response Theory analyses.

Item	Factor loading	Discrimination	Endorsability	UMS	WMS	S DIFF					
						USA vs Arg	USA vs Neth	USA vs Sp	Arg vs Neth	Arg vs Sp	Neth vs Sp
1	.765	.584	.148	.94	1.11	-.033	-.019	-.009	.001	.018	.018
2	.525	.392	.470	1.54	1.59	-.044	-.032	**-.073**	-.004	-.043	-.045
3	.719	.545	.208	1.07	1.34	.047	.029	.039	-.007	-.002	.006
4	.635	.501	.638	1.03	1.04	.019	**-.069**	.024	**-.086**	.015	**.090**
5	.912	.655	.080	.58	1.19	-.012	.007	.004	.011	.009	.000
6	.620	.493	.418	1.08	1.15	.006	-.032	.001	-.033	.002	.037
7	.870	.674	.102	.57	.96	-.019	-.013	-.022	-.007	-.011	.000
8	.793	.630	.189	.82	.97	.003	-.003	.008	-.003	.006	.008
9	.745	.599	.246	.95	1.00	.045	**.075**	.021	.029	-.027	**-.061**
10	.816	.651	.182	.67	.95	.009	-.004	.041	-.007	.035	.049
11	.748	.591	.235	.82	1.05	**-.070**	-.047	**-.066**	-.003	.004	.011
12	.643	.511	.388	1.13	1.20	.037	.028	**.056**	.005	.013	.022
13	.942	.688	.066	.42	.92	-.005	-.001	-.012	.006	-.003	-.005
14	.851	.676	.124	.62	.87	.001	.000	-.007	.006	-.009	-.016
15	.914	.672	.072	.40	.95	.013	.003	.004	-.006	-.008	.006
16	.942	.695	.067	.38	.97	-.004	-.002	.001	.002	.003	.005
17	.847	.688	.142	.52	.85	.007	.009	.007	.010	.002	-.010
18	.677	.542	.284	1.04	1.12	**.073**	.029	.049	-.031	-.017	.020
19	.626	.498	.337	1.10	1.04	-.018	-.015	-.028	.006	-.006	-.017
20	.499	.387	.704	1.31	1.24	.013	**-.068**	**-.053**	**-.083**	**-.064**	.012
21	.826	.616	.110	.74	.99	.009	.021	.010	.019	.002	-.021
22	.705	.549	.257	1.02	1.09	.002	**.054**	**.091**	**.057**	**.086**	.008
23	.488	.392	.752	1.29	1.18	-.040	.006	**-.059**	**.052**	-.019	**-.072**
24	.851	.661	.115	.69	1.00	.025	.008	**.051**	-.010	.027	.029
25	.883	.677	.093	.54	.87	-.005	-.011	.005	-.003	.011	.022
26	.592	.462	.348	1.24	1.32	-.023	-.026	**-.052**	-.006	-.024	-.027
27	.912	.680	.073	.49	.88	.011	.000	.013	-.009	-.001	.011
28	.933	.687	.067	.41	.94	.005	-.006	.003	-.006	-.001	.012
29	.750	.535	.159	1.23	1.43	-.039	-.034	-.045	-.016	-.012	.015
30	.972	.714	.055	.25	.86	-.002	.000	-.001	.000	.002	.000
31	.789	.603	.138	.85	1.09	.014	-.001	.023	-.011	.003	.027
32	.924	.666	.062	.50	.89	.007	.003	.009	-.007	.001	.009
33	.523	.411	.549	1.26	1.25	-.031	**.117**	-.022	**.145**	.014	**-.144**
34	.915	.669	.065	.49	.91	.005	-.005	-.003	-.008	-.006	.006
35	.906	.695	.082	.41	.90	-.003	-.001	-.008	-.002	-.002	-.001

*Note*. Highlighted items correspond to the final set of items included in the B-ABS scale. A full description of item content is provided in S1 Table. For the sPDIF results, values between .050 and .099 are shown in **bold** and values of .100 or higher are denoted in **bold and are underlined**.

### Classical Test Theory and Item Response Theory analyses

Table 1 presents the items’ performance based on the discrimination index, level of item endorsement, infit/outfit indices and sP-DIF. The results about the category characteristic curves and the item information curves of the ABS are presented in S1 Fig. Based on the previously indicated inclusion criteria, we selected items with an adequate discrimination index (between .43 and .63) with adequate infit/outfit values (UMS and WMS values between .50 and 1.50), and that showed sP-DIF < .05 across countries. Items 1, 3, 5, 6, 7, 8, 10, 14, 17, 19, 21, 25, 29, 31 and 32 fulfilled these criteria. However, item 3 (“I smoked cigarettes before the age of 14“) was excluded to avoid content and measures of drug use overlapping. Item 32 (“I have used illegal gadgets to make a telephone call”) was excluded because its meaning may not be clear for participants (i.e., it is ambiguous if the item referred to robbing a mobile, breaking the phone booths, etc.). Therefore, the final list includes items 1, 5, 6, 7, 8, 10, 14, 17, 19, 21, 25, 29, and 31.

### Internal consistency

Cronbach’s alphas for the whole sample (.890 [.884 .895]) and per country (USA: .910 [.904 .916]; Arg: .748 [.707 .785], Neth: .789 [.772 .806], Sp: .862 [.847 .877]) were significant at the 95% confidence interval (95%CI) and were higher than .70. Similar results were found with the ordinal omega at the 95%CI in the whole sample (.891 [.877 .904]) and per country (USA: .912 [.897 .923]; Arg: .751 [.672 .802], Neth: .817 [.681 .878], Sp: .863 [.827 .891]), which suggest adequate internal values consistency.

### Sources of criterion-related validity evidence

The descriptive results (i.e., mean and standard deviation) of the final 13-item version (B-ABS) and all the criterion variables for each country are presented in Table 2. We found higher antisocial behavior in Argentina, followed by Spain, USA and the Netherlands. However, these differences were small in magnitude. A large difference appeared between the Netherlands and the other countries (the Netherlands > USA Argentina, Spain) on the mean scores of the DSM-V Level 1 Measure psychosis domain. Additional mean differences were found in agreeableness (Argentina > USA), somatic distress (USA, Argentina, Spain > the Netherlands), sleep disturbances (Spain > the Netherlands) and marijuana frequency used in a typical week in the last 30 days (USA, Argentina > the Netherlands).

**Table 2:** Descriptive results.

	*n*				M (SD)				*d*					
	USA	Arg	Neth	Sp	USA	Arg	Neth	Sp	USA—Arg	USA—Neth	USA—Sp	Arg—Neth	Arg—Sp	Neth—Sp
**Big Five Personality domains**														
Emotional stability	1858	346	294	675	1.94 (.76)	1.89 (.84)	1.95 (.81)	2.03 (.78)	.06	-.01	-.12	-.07	-.17	-.10
Extraversion	1858	346	294	675	2.52 (.76)	2.45 (.78)	2.55 (.73)	2.55 (.74)	.06	-.04	-.04	-.13	-.13	.00
Openness	1858	346	294	675	2.67 (.63)	2.99 (.66)	2.69 (.63)	2.76 (.68)	.09	-.03	-.14	.*46*	.*34*	-.11
Agreeableness	1858	346	294	675	2.57 (.59)	2.71 (.58)	2.66 (.56)	2.71 (.61)	**-.50**	-.16	*-*.*22*	.09	.00	-.09
Conscientiousness	1858	346	294	675	2.48 (.64)	2.38 (.60)	2.33 (.67)	2.33 (.66)	*-*.*24*	.*23*	.*23*	.08	.08	.00
**Impulsivity traits**														
Negative urgency	1859	346	294	675	2.04 (.78)	2.03 (.71)	2.02 (.76)	2.13 (.68)	.16	.03	-.13	.01	-.14	-.15
Perseverance	1859	346	294	675	3.11 (.64)	2.95 (.63)	2.81 (.60)	3.02 (.64)	.01	.*48*	.13	.*23*	-.11	-.34
Premeditation	1859	346	294	675	3.19 (.62)	3.09 (.56)	2.97 (.61)	2.97 (.62)	.*25*	.*36*	.*33*	.*20*	.*20*	.00
Sensation seeking	1859	346	294	675	2.75 (.70)	2.47 (.81)	2.68 (.71)	2.57 (.75)	.17	.10	.*30*	*-*.*28*	-.13	.15
Positive urgency	1859	346	294	675	1.83 (.77)	1.55 (.65)	1.68 (.69)	1.76 (.71)	.*37*	.*21*	.01	-.19	*-*.*31*	-.11
**Mental health**														
Depression	1866	346	292	675	1.28 (1.06)	1.60 (1.03)	1.29 (.93)	1.61 (.92)	*-*.*31*	-.01	*-*.*33*	.*32*	-.01	*-*.*35*
Anger	1864	346	292	675	1.24 (1.09)	1.34 (1.14)	1.37 (.98)	1.37 (.99)	-.09	-.13	-.12	-.03	-.03	.00
Mania	1866	346	292	675	1.00 (.94)	1.09 (.89)	.98 (.85)	1.30 (.94)	-.10	.02	*-*.*32*	.13	*-*.*23*	*-*.*36*
Anxiety	1866	346	292	675	1.20 (1.04)	1.18 (.93)	.84 (.91)	1.07 (.83)	.02	.*37*	.14	.*37*	.12	*-*.*26*
Somatic distress	1866	346	292	675	.82 (1.00)	1.00 (1.00)	.31 (.78)	.87 (.87)	-.18	**.57**	-.05	**.77**	.14	**-.68**
Suicidal ideation	1865	346	292	675	.39 (.89)	.36 (.89)	.14 (.44)	.32 (.79)	.03	.*36*	.08	.*31*	.05	*-*.*28*
Psychosis	1866	346	292	675	.24 (.64)	.11 (.40)	.98 (1.09)	.17 (.49)	.*24*	**-.83**	.12	**-1.05**	-.13	**.96**
Sleep disturbance	1864	346	291	675	1.04 (1.21)	1.03 (1.23)	.56 (.96)	1.12 (1.15)	.01	.*44*	-.07	.*43*	-.08	**-.53**
Memory	1866	346	292	675	.63 (1.00)	.58 (.94)	.57 (.80)	.53 (.87)	.05	.07	.11	.01	.06	.05
Repetitive thoughts	1866	346	292	675	.57 (.87)	.61 (.90)	.25 (.63)	.47 (.79)	-.05	.*42*	.12	.*46*	.17	*-*.*31*
Dissociation	1857	346	292	675	.65 (1.03)	.63 (1.06)	.25 (.63)	.56 (.94)	.02	.*47*	.09	.*44*	.07	*-*.*39*
Personality functioning	1866	346	292	675	.98 (1.07)	1.00 (1.04)	.82 (.90)	1.00 (1.00)	-.02	.16	-.02	.19	.00	-.19
Alcohol use	1858	346	292	675	.48 (.84)	.46 (.75)	.66 (.83)	.33 (.67)	.03	*-*.*22*	.*20*	*-*.*25*	.18	.*44*
Tobacco use	1861	346	292	675	.38 (.91)	.60 (1.23)	.56 (1.15)	.64 (1.24)	*-*.*20*	-.17	*-*.*24*	.03	-.03	-.07
Illicit drug use	1861	346	292	675	.35 (.86)	.36 (.79)	.34 (.78)	.25 (.66)	-.01	.01	.13	.03	.15	.12
Total mental health score	1866	346	292	675	.80 (.66)	.86 (.56)	.75 (.49)	.83 (.51)	-.10	.09	-.05	.*21*	.06	-.16
**Marijuana outcomes**														
Marijuana status last 30 days	1214	250	157	437	.56 (.50)	.56 (.50)	.40 (.49)	.34 (.47)	.*39*	.*32*	.03	.*32*	.*45*	.12
Marijuana frequency last 30 days	1214	250	157	437	5.81 (9.26)	4.88 (8.10)	2.83 (6.68)	2.83 (6.83)	.00	.*37*	.*38*	.*28*	.*27*	.00
Marijuana quantity typical week	677	139	63	148	6.59 (11.23)	5.31 (9.68)	3.75 (8.61)	5.93 (12.42)	.11	.*28*	.08	.17	-.06	*-*.*20*
Marijuana frequency typical week	678	139	63	148	6.72 (8.93)	6.27 (6.42)	3.13 (4.31)	4.67 (6.03)	.12	**.51**	.*32*	**.57**	.*26*	*-*.*29*
Negative marijuana consequences	677	139	63	148	.17 (.20)	.17 (.20)	.16 (.17)	.22 (.20)	.00	.05	*-*.*35*	.05	*-*.*25*	*-*.*32*
**Antisocial behavior**	1873	346	294	675	1.17 (.34)	1.16 (.20)	1.11 (.20)	1.20 (.28)	.03	.*22*	-.10	.*25*	-.16	*-*.*37*

*Note*. Low mean differences are *italicized* (.20 < *rdiff* < .50), medium differences are shown in **bold** (.50 < *rdiff* < .80), and large difference are denoted in **bold and are underlined** (*d* > .80) (Cohen, 1992).

Table 3 presents Pearson’s correlations between antisocial behavior and the criterion-related variables. Across countries, antisocial behavior was statistically significantly associated with low agreeableness, low conscientiousness, negative urgency, lack of premeditation, positive urgency, mania, somatic distress, suicidal ideation, psychosis, memory problems, repetitive thoughts, dissociation, personality functioning, tobacco use, illicit drug use, poor mental health, and negative marijuana-related consequences. In some, but not all the countries, antisocial behavior also showed significant associations with lack of perseverance (USA and Spain), sensation seeking (Argentina and Spain), depression (USA and the Netherlands), anger (USA and Argentina), anxiety (USA, Spain and the Netherlands), sleep disturbance (USA, Argentina and the Netherlands), alcohol use (USA, Spain and Argentina), frequency of marijuana use (USA>, Spain and Argentina) and quantity of marijuana use (USA, and Argentina).

**Table 3:** Correlations between the B-ABS and the criterion variables and correlation differences across countries.

	*r* with antisocial behavior				Correlation differences					
	USA	Arg	Neth	Sp	USA—Arg	USA—Neth	USA—Sp	Arg—Neth	Arg—Sp	Neth—Sp
**Big 5 PD**										
Emotional stability	-.023	-.003	.066	-.030	.020	.089	.007	.069	.027	.096
Extraversion	**-.096**	.058	.011	-.002	.*154*	.107	.094	.047	.060	.013
Openness	**-.142**	-.028	-.052	-.072	.114	.090	.070	.024	.044	.020
Agreeableness	**-.280**	**-.190**	**-.247**	**-.246**	.090	.033	.034	.057	.056	.001
Conscientiousness	**-.246**	**-.209**	**-.167**	**-.220**	.037	.079	.026	.042	.011	.053
**Impulsivity traits**										
Negative urgency	**.190**	**.266**	**.204**	**.152**	.076	.014	.038	.062	.114	.052
Perseverance	**-.131**	-.009	-.059	**-.161**	.122	.072	.030	.050	.*152*	.102
Premeditation	**-.267**	**-.144**	**-.167**	**-.224**	.123	.100	.043	.023	.080	.057
Sensation seeking	.026	**.294**	.111	**.115**	**.268**	.085	.089	.*183*	.*179*	.004
Positive urgency	**.257**	**.210**	**.314**	**.269**	.047	.057	.012	.104	.059	.045
**Mental health symptoms**										
Depression	**.106**	**.060**	**.154**	.037	.046	.048	.069	.094	.023	.117
Anger	**.103**	**.116**	.077	.046	.013	.026	.057	.039	.070	.031
Mania	**.211**	**.142**	**.128**	**.101**	.069	.083	.110	.014	.041	.027
Anxiety	**.124**	**.096**	**.123**	**.094**	.028	.001	.030	.027	.002	.029
Somatic distress	**.191**	**.196**	**.148**	**.119**	.005	.043	.072	.048	.077	.029
Suicidal ideation	**.253**	**.154**	**.237**	**.183**	.099	.016	.070	.083	.029	.054
Psychosis	**.370**	**.236**	**.293**	**.310**	.*134*	.077	.060	.057	.074	.017
Sleep disturbance	**.136**	**.131**	**.136**	.074	.005	.000	.062	.005	.057	.062
Memory	**.228**	**.194**	**.318**	**.207**	.034	.090	.021	.124	.013	.111
Repetitive thoughts	**.277**	**.203**	**.190**	**.252**	.074	.087	.025	.013	.049	.062
Dissociation	**.235**	**.142**	**.331**	**.177**	.093	.096	.058	**.189**	.035	.*154*
Personality functioning	**.167**	**.122**	**.219**	**.171**	.045	.052	.004	.097	.049	.048
Alcohol use	**.299**	**.147**	.034	**.266**	.*152*	**.265**	.033	.113	.119	**.232**
Tobacco use	**.326**	**.191**	**.235**	**.191**	.*135*	.091	.*135*	.044	.000	.044
Illicit drug use	**.326**	**.201**	**.243**	**.232**	.125	.083	.094	.042	.031	.011
Total mental health score	**.294**	**.242**	**.305**	**.251**	.052	-.011	.043	.063	.009	.054
**Marijuana outcomes**										
Marijuana status last 30 days	**.083**	.092	.023	**.180**	.009	.060	.097	.069	.088	.*157*
Marijuana frequency last 30 days	**.094**	**.205**	.099	**.153**	.111	.005	.059	.106	.052	.054
Marijuana quantity typical week	**.132**	**.247**	-.038	.026	.115	.*170*	.106	**.285**	**.221**	.064
Marijuana frequency typical week	.054	**.248**	.226	.070	**.194**	.*172*	.016	.022	.*178*	.*156*
Negative marijuana consequences	**.137**	**.293**	**.351**	**.166**	.*156*	**.214**	.029	.058	.127	**.185**

*Note*. USA = United States; Arg = Argentina; Neth = Netherlands; Sp = Spain. Correlations with a *p* < .05 or lower are marked in bold. Medium correlation differences are *italicized* (.128 < *rdiff* < .184), large differences are shown in **bold** (.184 < *rdiff* < .240), and substantial differences are denoted in **bold and are underlined** (*rdiff* > .240).

The magnitude of the differences in the correlation coefficients across countries is also presented in Table 3. Across 186 possible comparisons, we found that the average difference in correlations was .072 (*SD* = .056). We considered differences < 1 *SD* to be small, between 1 *SD* and 2 *SD* were considered medium, between 2 *SD* and 3 *SD* were taken as being large, and differences above 3 *SD* were considered substantial. We found substantial differences between antisocial behavior and: a) sensation seeking between USA and Argentina, with a nonsignificant correlation in the USA (*r* = .026, *p* >.05) and a positive medium-size correlation in Argentina (*r* = .294, *p* < .01); b) alcohol use between the USA and the Netherlands, with a positive medium-size correlation in the USA (*r* = .299, *p* < .001) and a nonsignificant correlation in the Netherlands (*r* = .034, *p* >.05); c) marijuana quantity used in a typical week in the last 30 days between Argentina and Netherlands, with a positive medium-size correlation in Argentina (*r* = .247, *p* < .01) and a negative and nonsignificant correlation in the Netherlands (*r* = -.038, *p* >.05).

## Discussion

The present research aimed to adapt the current version of the Spanish ABS scale to English and Dutch, and create a Brief version of the measure which could be particularly useful and necessary when administration time is limited. Following the international guidelines for test adaptations [35], after detecting the lack of measures to assess antisocial behavior in youths cross-nationally, an iterative adaptation of the ABS to the different languages was performed. Subsequently, confirmation guidelines were followed and evidence of the structure of the measure was provided, as the equivalence of the measure across countries, reliability of the scores and sources of criterion-validity evidences of the brief measure [35].

Regarding the structure of the ABS, we found evidence for the unidimensional structure underlying the set of items. To identify a brief set of items, without diminishing the validity and reliability properties, we followed a multi-prong approach. As previously recommended [52], we combined analyses from the Classical Test Theory and the Item Response Theory. The resulting brief scale (B-ABS) comprised 13 high-quality items that exhibited salient factor loadings, adequate discrimination, adequate values for infit/outfit indices, variability in its endorsability, and similar item functioning across different countries/cultures. The B-ABS showed adequate Cronbach’s alpha and ordinal omega indices in the four countries based on the standard cut-offs, which provide evidence of the reliability of the scores of the short version of the ABS.

Regarding sources of content validity evidence of the resulting B-ABS, it seems that the B-ABS is a more face valid measure of antisocial behavior understood as “actions that violate societal norms and others’ personal or property rights”. While the original SRD contains items like “I saw pornographic films before the age of fourteen” (item 2), “I smoked cigarettes before the age of fourteen” (item 3), “I drunk alcohol before the age of fourteen” (item 12), or “I have taken some type of drugs” (item 20), with a content that is difficult to justify as antisocial behavior, the B-ABS contains more pure antisocial behaviors and reflect the interpersonal component of the antisocial behavior construct. In addition, the B-ABS does not contain explicit personality content. Scales like the PCL-R [24], Antisocial Processes Screening Device [23], and other popular measures [e.g., Buss-Perry Aggression Questionnaire, 54] that assess antisocial behavior contain a good amount of trait content. Thus, if one is more interested in assessing antisocial behavior per se, the B-ABS has the advantage of not inflating effect sizes in particular research contexts due to criterion-predictor overlap.

When the antisocial behavior scores obtained in the B-ABS were compared across countries, we found higher antisocial behavior in Argentina, followed by Spain, USA and the Netherlands. These results contrast with the hypothesis than individualistic countries (e.g., USA and the Netherlands) have higher antisocial behavior than the collectivistic ones (e.g., Argentina and Spain) [15, 16]. In addition, it is partially supported that income inequality hypothesis could enhance antisocial behavior [17, 18], as the sample of youths from Netherlands, the country with lower income inequality [based on the Gini index of the World Bank estimate, 55], showed the lowest antisocial behavior rate while Argentina, the country with higher income inequality, showed the highest antisocial behavior score. However, it is important to note that the mean differences in the mean levels of antisocial behavior between the Netherlands and the rest of the countries were low, and that the sample size of the Netherlands was the smallest one in the study. Thus, additional studies should further explore if the differences in the income inequality are related or not with the higher/lower rates of antisocial behavior across countries.

Consistent with previous meta-analyses, the highest correlations between the B-ABS and personality traits were found with (low) agreeableness and (low) conscientiousness [28–30]. When we focused on specific impulsivity facets, we found that negative and positive urgency and premeditation were significantly related to antisocial behavior in all countries, while perseveration correlated with antisocial behavior in the USA and Spain, and sensation seeking in Argentina and Spain. Previous studies have also found that these five impulsivity facets are related to different aspects of antisocial behavior, such as aggression, rule-breaking behavior, theft and vandalism [56, 57], and that a genetic overlap occurs between these impulsivity facets and antisocial behavior [31]. The only substantial difference between the correlations of S UPPS-P and B-ABS was found with the sensation seeking subscale, which showed a nonsignificant correlation in the USA, and significant and positive medium-sized correlation in Argentina. Although the nonsignificant association in the USA sample was somewhat unexpected [31], there are reasons that might explain this result. For instance, sensation seeking displays an age-related pattern and peaks in teenage years and the early twenties [58, 59]. Notably, the difference in the mean age between the Argentinean (*M* = 24.31) and USA students (*M* = 19.97) was the biggest across all four countries.

Regarding the association of antisocial behavior with different mental health outcomes, we found that antisocial behavior correlated generally with externalizing (i.e., alcohol, tobacco and illicit drug use) and internalizing symptoms (i.e., depression, anxiety and somatic distress), which corroborates previous studies [60]. These associations were slightly higher with the externalizing than with the internalizing symptoms, suggesting that antisocial behavior and substance use are well conceptualized as part of the same spectrum [61]. One unexpected result was that alcohol use was not related to antisocial behavior in the Netherlands and, consequently, substantial differences in the magnitude of the correlations between antisocial behavior and alcohol use were found between USA (with positive medium-sized correlations) and the Netherlands. Future research is needed to corroborate these findings.

Previous studies have found that antisocial behavior is related to sleep problems [62], suicidal behavior [63], or depression symptoms [9]. The current research adds evidence to these findings by relating antisocial behavior with these domains and a wide variety of health outcomes in four different countries.

Antisocial behavior was also related to some marijuana outcomes in the four countries, and this result is consistent with studies reporting that individuals with conduct problems are at increased risk for substance (ab)use, including marijuana [64]. However, a substantial difference in the magnitude of the correlation of antisocial behavior with quantity of marijuana use appeared between Argentina and the Netherlands. Large differences in the magnitude of other correlations between antisocial behavior and negative marijuana consequences emerged between the Netherlands and the USA and Spain. However, these results should be taken with caution because the sample size of the Dutch marijuana users was small (*n* = 63).

### Limitations

The present findings should be interpreted by considering a number of limitations. First, participants were recruited by different strategies (e.g., email listings, posting an invitation on online social networks, pool of students) across countries and different incentives for participation we also employed (e.g., raffles with prizes, research participation credit and money). These issues potentially limit generalizability to other youth samples. Second, we reported preliminary evidence of the structure and the invariance across countries of the resulting B-ABS. Thus, evidence of the adequacy of the structure of the B-ABS should be replicated in an independent cross-national sample performing Confirmatory Factor Analysis [65]. Third, our sample comprise a higher percentage of women than men. Considering than men usually report higher antisocial behavior than women [4], a higher representation of men in our sample would be advisable. Fourth, to avoid the burden produced by a long assessment protocol, only sex and age were assessed across countries. However, the inclusion of other demographic data could have provided a better description of the cross-national sample. Fifth, the sample size differed across sites, and was low in the case of the drug consumers of the Netherlands. Thus, we may have been underpowered to detect smaller differences in mean differences/correlations across cultures. Sixth, previous cross-national research has also demonstrated different levels of social desirability related to admitting antisocial behavior across countries [66]. Thus, objective information to assess antisocial behavior (e.g., criminal history) or infrequency and desirability scales could help to better reflect the similarities/differences in antisocial behavior among the samples of youths included in the present research. In any case, it is also remarkable that, although the higher/lower social desirability across countries could influence the prevalence of the reported behaviors, the self-reports have also demonstrated to be appropriate and valid to test theoretical correlates cross-nationally [66]. Seventh, although this brief measure provides an adequate assessment of antisocial behavior in college students from four countries, this version may not be appropriate for other college students or non-college students. Validation for Spanish-, English- and Dutch-speakers from other parts of the world will be an important next step. Finally, although we selected the best set of items following multiple quality criteria, we did not examine the association of this brief version with other (brief) measures of antisocial behavior.

## Conclusion

The present study offers preliminary sources of reliability and validity evidence of the B-ABS to measure antisocial behavior. The results from both the Classical Test Theory and Item Response Theory offer encouraging evidence for the psychometric properties of a 13-item version of the measure and suggest that it can be used to assess antisocial behaviors in college students from different countries. Furthermore, cross-national comparisons of correlates of antisocial behavior offer avenues for future research to target the key risk factors associated with antisocial behavior. Overall, our findings suggest that the 13-item B-ABS scores represented a valid and reliable alternative to efficiently measure antisocial behaviors in young adults from different countries/cultures.

## Supporting information

S1 Table ABS instructions and items in English, Dutch and Spanish. (DOCX)

S1 Fig The item response category characteristic curves and item information curves of the ABS. The final 13 B-ABS items are marked in black squares.
(DOCX)
