# Supplementary material for: Preliminary validity and reliability evidence of the Brief Antisocial Behavior Scale (B-ABS) in young adults from four countries
Source: PLoS One. 2021 Feb 22;16(2):e0247528. doi: 10.1371/journal.pone.0247528 (PMC7899364; doi:10.1371/journal.pone.0247528)
Supplement: S1 Table — (DOCX) [file pone.0247528.s001.docx]

**S1 Table. ABS instructions and items in English, Dutch and Spanish.** The final pool of items of the Brief SRAB-R are marked by an asterisk.

Read each one of the following sentences and indicate the frequency in which you have done these behaviors.

Lees elk van de volgende uitspraken en geef aan of u zich aan dat gedrag hebt schuldig gemaakt.

Por favor, lee cada una de las siguientes frases e indica la frecuencia con la que realizaste cada una de estas conductas

| Never or almost never  Nooit of bijna nooit  Nunca o casi nunca | Sometimes  Soms  Algunas veces | Frequently  Vaak  Frecuentemente | Very Frequently  Zeer vaak  Muy frecuentemente |
| --- | --- | --- | --- |
| 1 | 2 | 3 | 4 |

| Item  No. | Language | Content |
| --- | --- | --- |
| 1* | English | I have stolen equipment or money from slot, game, electronic or vending machines (e.g., tobacco, snacks) |
|  | Dutch | Ik heb apparatuur of geld gestolen uit slot-, spel-, elektronische of automaten (bijvoorbeeld sigaretten, snacks) |
|  | Spanish (Arg) | He robado materiales o dinero de máquinas tragamonedas, electrónicas, o expendedoras (e.g., cigarrillos, golosinas, gaseosas) |
|  | Spanish (Cst) | He robado materiales o dinero de máquinas de juego o tragaperras, electrónicas, o expendedoras (e.g., tabaco, snacks) |
| 2 | English | I saw pornographic films before the age of fourteen |
|  | Dutch | Ik keek pornografische films voor mijn veertiende levensjaar |
|  | Spanish | Vi películas pornográficas antes de los catorce años |
| 3 | English | I smoked cigarettes before the age of fourteen |
|  | Dutch | Ik rookte sigaretten voor mijn veertiende levensjaar |
|  | Spanish | Fumé cigarrillos antes de los catorce años |
| 4 | English | I have trespassed on rail tracks, industrial estates, private gardens, empty houses, factories, etc. |
|  | Dutch | Ik heb inbreuk gemaakt op spoorpaden, industrieterreinen, privétuinen, lege huizen, fabrieken, etc |
|  | Spanish | He pasado por lugares donde está prohibido el paso (vías de ferrocarril, zonas de mercancías, jardines privados, casas vacías, fábricas, etc.) |
| 5* | English | I have taken a vehicle (bicycle, motorbike, car) from someone else and kept it |
|  | Dutch | Ik heb een voertuig (fiets, motorfiets, auto) van iemand anders meegenomen en gehouden |
|  | Spanish (Arg) | He tomado un vehículo (bicicleta, motocicleta, auto) de otra persona y me lo he quedado |
|  | Spanish (Cst) | He cogido un vehículo (bicicleta, motocicleta, coche) de otra persona y me lo he quedado |
| 6* | English | I have forged signatures, medical prescriptions or other documents |
|  | Dutch | Ik heb handtekeningen, medische voorschriften of andere documenten vervalst |
|  | Spanish | He falsificado firmas, recetas médicas u otros documentos |
| 7* | English | I have struggled or fought to get away from the police |
|  | Dutch | Ik heb verzet gepleegd of gevochten om weg te komen van de politie |
|  | Spanish | He forcejeado o peleado para escapar de algún policía |
| 8* | English | I have bought or accepted as presents cheap goods that I knew had been stolen |
|  | Dutch | Ik heb cadeautjes als goedkope goederen gekocht of geaccepteerd waarvan ik wist dat ze gestolen waren |
|  | Spanish | He comprado de forma barata o he aceptado regalos que sabía que eran robados |
| 9 | English | I have deliberately scattered trash on the pavement, broken bottles on the floor, etc. |
|  | Dutch | Ik heb opzettelijk afval verspreid op de trottoir, gebroken flessen op de vloer, etc. |
|  | Spanish (Arg) | He tirado deliberadamente en la vereda basura, he roto botellas en el suelo, etc. |
|  | Spanish (Cst) | He esparcido deliberadamente en la acera basuras, he roto botellas en el suelo, etc. |
| 10* | English | I have stolen things from newsstands or corner shops |
|  | Dutch | Ik heb dingen gestolen uit krantenkiosken of winkels |
|  | Spanish | He robado cosas de kioscos o pequeñas tiendas |
| 11 | English | I have shoplifted from department stores, supermarkets or chain stores |
|  | Dutch | Ik heb winkeldiefstal gepleegd in warenhuizen, supermarkten of winkelketens |
|  | Spanish | He robado objetos de grandes almacenes, supermercados u otras tiendas grandes |
| 12 | English | I drunk alcohol before the age of fourteen |
|  | Dutch | Ik dronk alcohol voor mijn veertiende |
|  | Spanish | Tomé bebidas alcohólicas antes de los catorce años |
| 13 | English | I have used blunt objects or a knife in some fights |
|  | Dutch | Ik heb als ik vocht stompe objecten of een mes gebruikt |
|  | Spanish | He usado algún objeto contundente o navaja en alguna pelea |
| 14* | English | I have broken windows and glass doors |
|  | Dutch | Ik heb ramen en/of glazen deuren gebroken |
|  | Spanish | He roto cristales de ventanas y puertas |
| 15 | English | I have robbed an enemy or someone from a rival gang |
|  | Dutch | Ik heb een vijand of iemand van een rivaliserende bende beroofd |
|  | Spanish | He atacado a algún enemigo o a alguien de una banda rival |
| 16 | English | I have stolen objects from cars |
|  | Dutch | Ik heb voorwerpen uit auto's gestolen |
|  | Spanish | He robado objetos de los coches |
| 17* | English | I have broken, scratched or damaged things of public use (in streets, cinemas, dance halls, railway carriages, buses, etc.) |
|  | Dutch | Ik heb dingen gebroken, bekrast of beschadigd van openbaar gebruik (in straten, bioscopen, danszalen, spoorwegwagons, bussen, enz.) |
|  | Spanish | He roto, rasgado o dañado cosas de uso público (en calles, cines, bailes, vagones de trenes, autobuses, etc.) |
| 18 | English | I have taken money from home with no intention of returning it |
|  | Dutch | Ik heb geld van thuis meegenomen zonder de intentie om het terug te geven |
|  | Spanish | He tomado dinero de casa sin intención de devolverlo |
| 19* | English | I have annoyed, insulted or fought with strangers |
|  | Dutch | Ik heb vreemden geïrriteerd, beledigd of met ze gevochten |
|  | Spanish | He molestado, insultado o me he peleado con gente desconocida |
| 20 | English | I have taken some type of drugs |
|  | Dutch | Ik heb verschillende soorten drugs gebruikt |
|  | Spanish | He consumido algún tipo de drogas |
| 21* | English | I have paid with fake coins or paid less than the bus, train or metro fare |
|  | Dutch | Ik heb met vals geld betaald of minder dan de origineelprijs betaald voor de bus, trein of metro |
|  | Spanish | He pagado con monedas falsas o menos de lo que costaba en algún autobús, tren o metro |
| 22 | English | I have deliberately sneaked onto the bus, train or subway |
|  | Dutch | Ik ben opzettelijk in de bus, trein of metro geslopen om zwart te rijden |
|  | Spanish (Arg) | Me he colado deliberadamente sin pagar en el colectivo, tren o metro |
|  | Spanish (Cst) | Me he colado deliberadamente sin pagar en el autobús, tren o metro |
| 23 | English | I played hooky from school |
|  | Dutch | Ik heb gespijbeld van school |
|  | Spanish (Arg) | Falté o me escapé de la escuela |
|  | Spanish (Cst) | Hice novillos en la escuela |
| 24 | English | I have gone out with a gang on a spree that ended up in a fight |
|  | Dutch | Ik ben uitgegaan met een bende om lol te trappen dat eindigde in een gevecht |
|  | Spanish (Arg) | He ido en grupo haciendo lío que ha desembocado en alguna pelea |
|  | Spanish (Cst) | He ido en grupo haciendo juerga que ha desembocado en alguna pelea |
| 25* | English | I have scratched cars or broken rear view mirrors |
|  | Dutch | Ik heb auto's bekrast of achteruitkijkspiegels gebroken |
|  | Spanish (Arg) | He rayado la chapa o he roto los espejos de coches |
|  | Spanish (Cst) | He rayado la chapa o he roto los retrovisores de coches |
| 26 | English | I drove a car, motorbike or scooter before the authorized age |
|  | Dutch | Ik heb als minderjarige een auto, motorfiets of scooter gereden |
|  | Spanish (Arg) | Conduje algún auto, ciclomotor, motocicleta o vespa antes de la edad autorizada |
|  | Spanish (Cst) | Conduje algún coche, ciclomotor, motocicleta o vespa antes de la edad autorizada |
| 27 | English | I have taken a car, motorbike or bike from a stranger (without the intention of stealing it) just for fun |
|  | Dutch | Ik heb een auto, motorfiets of fiets van een vreemdeling (zonder de bedoeling om deze te stelen) gewoon voor de lol meegenomen |
|  | Spanish (Arg) | He tomado algún coche, ciclomotor o bicicleta de alguna persona desconocida (sin intención de robarlo) sólo por divertirme |
|  | Spanish (Cst) | He cogido algún coche, ciclomotor o bicicleta de alguna persona desconocida (sin intención de robarlo) sólo por divertirme |
| 28 | English | I have forced the lock on a department store, garage, warehouse or pavilion door |
|  | Dutch | Ik heb het slot gebroken van een warenhuis, garage, magazijn of paviljoen deur |
|  | Spanish (Arg) | He forzado la puerta de algún gran almacén, garaje, almacén o galpón |
|  | Spanish (Cst) | He forzado la puerta de algún gran almacén, garaje, almacén o pabellón |
| 29* | English | I have carried weapons (such as knives or clubs) in case I needed them in a fight |
|  | Dutch | Ik heb wapens (zoals messen of knuppels) met me mee gedragen voor het geval ik ze nodig had in een gevecht |
|  | Spanish (Arg) | He llevado encima algún tipo de arma blanca (como navajas) para el caso de necesitarla en alguna pelea |
|  | Spanish (Cst) | He llevado encima algún tipo de arma blanca (como navajas o porras) para el caso de necesitarla en alguna pelea |
| 30 | English | I have broken into a house or flat to rob |
|  | Dutch | Ik heb ingebroken in een huis of flat om deze leeg te halen |
|  | Spanish (Arg) | He entrado en alguna casa o departamento para robar |
|  | Spanish (Cst) | He entrado en alguna casa o piso para robar |
| 31* | English | I have spray painted walls, traffic signs or pointing signs |
|  | Dutch | Ik heb graffiti gesprayd op geverfde muren, verkeersborden of borden |
|  | Spanish (Arg) | He pintado alguna pared, señal de tráfico o señales indicativas con un “aerosol” |
|  | Spanish (Cst) | He pintado alguna pared, señal de tráfico o señales indicativas con un “espray” |
| 32 | English | I have used illegal devises to make telephone calls |
|  | Dutch | Ik heb illegale apparaten gebruikt om telefoontjes te plegen |
|  | Spanish | He realizado acciones ilegales para llamar por teléfono |
| 33 | English | I have infringed traffic laws (e.g., going through a red light, jumping a stop sign, etc.) |
|  | Dutch | Ik heb verkeerswetten overtreden (bijvoorbeeld door een rood licht gereden, een stopteken negeren, etc.) |
|  | Spanish (Arg) | He violado las normas de tránsito (pasar semáforos rojos, no parar en paradas, etc.) |
|  | Spanish (Cst) | He infringido el código de circulación (saltar semáforos rojos, no parar en los stops, etc.) |
| 34 | English | I have mistreated animals |
|  | Dutch | Ik heb dieren mishandeld |
|  | Spanish | He maltratado animales |
| 35 | English | I have made money from drug trafficking |
|  | Dutch | Ik heb geld verdiend aan drugshandel |
|  | Spanish | He conseguido dinero mediante el tráfico de drogas |
