# Supplementary material for: Preliminary validity and reliability evidence of the Brief Antisocial Behavior Scale (B-ABS) in young adults from four countries
Source: PLoS One. 2021 Feb 22;16(2):e0247528. doi: 10.1371/journal.pone.0247528 (PMC7899364; doi:10.1371/journal.pone.0247528)

**S1 Fig. The Item Response Category Characteristic Curves and Item Information Curves of the ABS.** The Final 13 B-ABS Items are Marked in Black Squares.


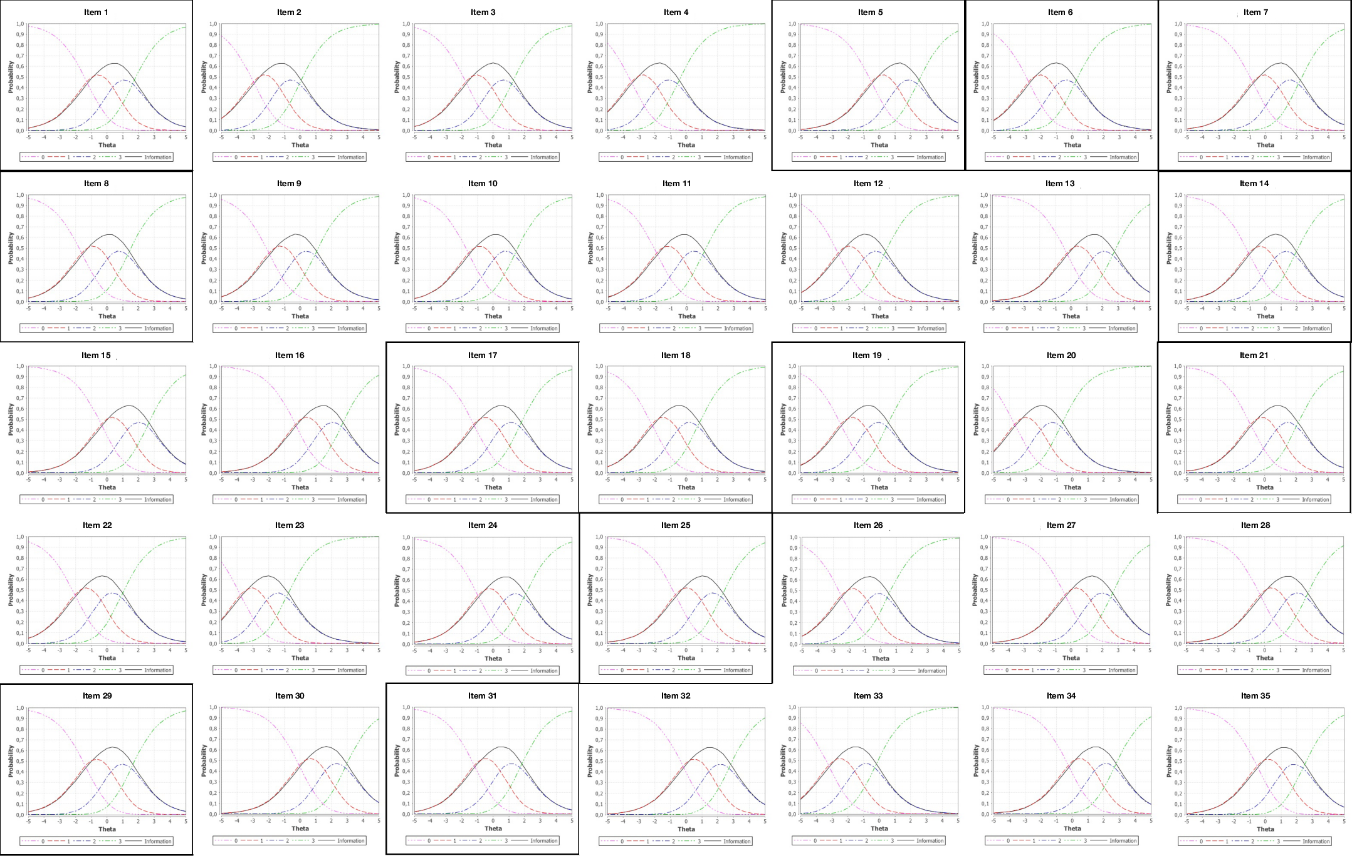

Supplement: S1 Fig — The final 13 B-ABS items are marked in black squares. (DOCX) [file pone.0247528.s002.docx]
